# Fluorescence visualization for cancer DETECTION: EXPERIENCE and perspectives

**DOI:** 10.1016/j.heliyon.2024.e24390

**Published:** 2024-01-09

**Authors:** Yaroslav Kravchenko, Kateryna Sikora, Andrew Awuah Wireko, Mykola Lyndin

**Affiliations:** a Sumy State University, Sumy, Ukraine; b NanoBioMedical Centre, Adam Mickiewicz University in Poznan, Poznan, Poland; c Institute of Anatomy, Medical Faculty, University of Duisburg-Essen, Essen, 45147, Germany

**Keywords:** Intraoperative fluorescence diagnosis, Fluorophore-conjugated antibodies, Fluorescent probes, Fluorescent labels, Cancer diagnostic

## Abstract

The current review focuses on the latest advances in the improvement and application of fluorescence imaging technology. Near-infrared (NIR) fluorescence imaging is a promising new technique that uses non-specific fluorescent agents and targeted fluorescent tracers combined with a dedicated camera to better navigate and visualize tumors. Fluorescence-guided surgery (FGS) is used to perform various tasks, helping the surgeon to distinguish lymphatic vessels and nodes from surrounding tissues easily and quickly assess the perfusion of the planned resection area, including intraoperative visualization of metastases. The results of the insertion of fluorescence visualization as an auxiliary method to cancer detection and high-risk metastatic lesions in clinical practice have demonstrated enthusiastic results and huge potential. However, intraoperative fluorescence visualization must not be considered as a main diagnostic or treatment method but as an aid to the surgeon. Thus, fluorescence study does not dispense the diagnostic gold standards of benign or malignant tumors (conventional examination, biopsy, ultrasonography and computed tomography, etc.) and can be done usually during intraoperative treatment. Moreover, as fluorescence surgery and fluorescence diagnostic techniques continue to improve, it is likely that they will evolve towards targeted fluorescence imaging probes that will increasingly target a specific type of cancer cell. The most important point remains the search for highly selective messengers of fluorescent labels, which make it possible to identify tumor cells exclusively in the affected organs and indicate to surgeons the boundaries of their spread and metastasis.

## 1 Introduction

The arsenal of tools and assistive technologies of modern surgery is expanding at an accelerated pace. Over the past few decades, magnetic resonance imaging, computed tomography, positron emission tomography, and other methods have appeared among such imaging “tools”.The above techniques and their widespread use in routine diagnostics have significantly improved the statistics of early cancer detection and obtaining negative tumor resection margins without requiring repeated surgical intervention [[1], [2], [3]]. However, ensuring a tumor-free excision margin during surgery remains an urgent problem in clinical oncology.

Intraoperative frozen section analysis is widely used to determine tumor margins during excision. However, this method is time-consuming (up to 20 min if all goes well) and requires well-trained staff [4,5]. Even with the well-coordinated work of the surgeon and the pathologist, this method should not be considered a complete replacement for the paraffin embedding of samples. The frozen section method has many limitations compared to paraffin-embedded tissue sections due to the possible poor quality of the obtained samples and insufficient tissue staining. The paper [6] studied the accuracy and features of the diagnosis of frozen sections of primary ovarian tumors and ovarian metastases. Among 802 primary ovarian tumors examined, 50 cases (6.2 %) presented conflicting diagnoses, with frequent misinterpretations observed for mucinous carcinoma (40.5 %), low-grade serous carcinoma (LGSC; 31.3 %), and mucinous borderline tumor (18.4 %). Notably, all four instances of low-grade appendicular mucinous neoplasms among the 69 ovarian metastases were incorrectly diagnosed as primary ovarian mucinous tumors. This investigation has successfully identified histological subtypes of both primary ovarian tumors and ovarian metastases from other cancers that are susceptible to misdiagnosis during intraoperative frozen section analysis.

Other diagnostic methods to achieve negative resection margins include visual inspection and palpation. The latter makes it impossible to detect small tumors. Palpation has limited sensitivity and is increasingly out of use due to the increasing use of robotic laparoscopic surgery. A relatively new trend is fluorescent interoperative imaging, a non-contact, relatively cheap, and non-ionizing navigation technique that is being actively implemented in surgical practice [7,8].

**Fig. 1:**
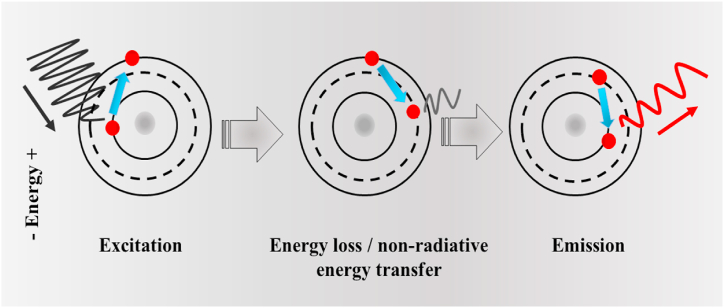
Schematic drawing demonstrating the principle of fluorescence.

To begin with, let's briefly recall what the phenomenon of fluorescence is and what physical principles it is based on. The physicist George Stokes first observed the fluorescence of quinine compounds in 1852. Fluorescence is the emission of light by a substance that has absorbed light or other electromagnetic radiation. According to the concepts of quantum chemistry, electrons in atoms are located at energy levels. The energy difference between the energy levels and the frequency of oscillations of the absorbed light is related to each other. Following the absorption of light, a portion of the energy assimilated by the system is dissipated through relaxation processes. Subsequently, a fraction of this energy may be released as a photon with specific energy (refer to Fig. 1) [9]. When the organic molecules of the fluorochrome capture light energy, electrons in a delocalized state undergo promotion from a ground state to a higher energy level. As these electrons transition from the excited singlet state back to the ground state, the released energy manifests as photons, which are then detected as fluorescence by the observer's eye. A singlet state usually refers to a system in which all electrons are paired. A singlet state typically denotes a system wherein all electrons are paired. The term “singlet” signifies a cohesive assembly of particles with a collective angular momentum of zero, and a total spin quantum number s = 0. Fluorescence photons exhibit lower energy (hv_*em*_) in comparison to the photons utilized to induce the excited state (hv_*ex*_). The excited state S1 has alternative relaxation mechanisms, not entailing light emission, which are referred to as non-radiative processes. These processes compete with fluorescence emission, thereby diminishing its efficiency. Internal conversion elucidates the portion of absorbed energy that did not transform into fluorescent radiation.

**Fig. 2:**
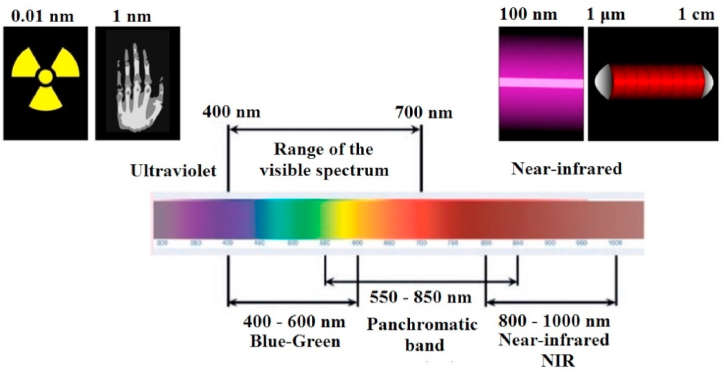
Electromagnetic spectrum with a detailed description of the visible and NIR wavelength ranges [adapted from 9].

However, when getting acquainted with the current technologies of fluorescent surgery, we will operate with such quantities as the wavelength of excitation and fluorescence. The fluorescence spectrum is shifted relative to the absorption spectrum towards long wavelengths. This phenomenon is called the Stokes shift caused by nonradiative relaxation processes. As a result, part of the energy of the absorbed photon is lost, and the emitted photon has lower energy, and, accordingly, a longer wavelength [[10], [11], [12]]. Fig. 2 shows the spectrum of electromagnetic radiation with a detailed description of the visible and infrared (IR) ranges.

Although the literature describes cases of combining antibodies labeled with radionuclide and fluorophore [13], our review focuses on nonionizing navigation. However, we would still like to briefly acquaint the reader with the achievements in optimizing labeled antibodies with a radionuclide (so-called radioactive label) and a double label. Double labeling of the tumor lesion results to a high tumor/background signal ratio [14]. Although sensitivity is relatively high (in the range of 10^− 9^ to 10^−^^12^ M), the doses of fluorescent agents needed for imaging are considerably larger than those used for nuclear imaging.

## 2 Clinically intraoperative available fluorescence imaging techniques

**Fig. 3:**
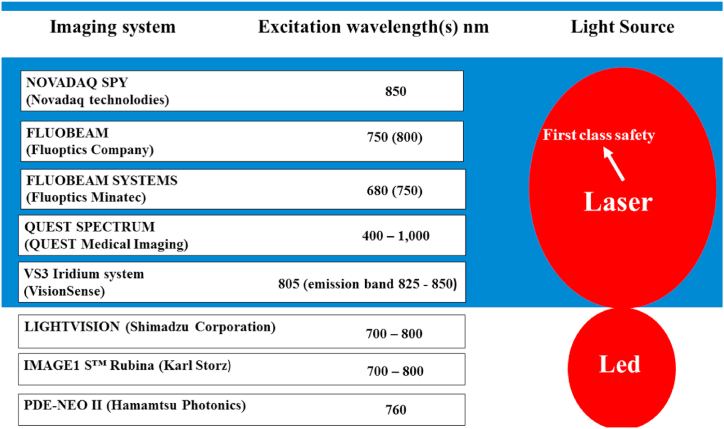
Clinically available fluorescence imaging systems and some of their characteristics.

Currently, most of the images for intraoperative navigation are obtained using the Novadaq SPY system (approved by the Federal Drug Administration (FDA) in 2005). Novadaq Spy can display a fluorescent contrast agent that emits light at a wavelength of 830 nm as it passes through the vascular bed or myocardial chambers. The passage of the contrast agent can be observed in real-time. The market for systems for fluorescent oncological surgery is expanding every year. On Fig. 3 shows several new FDA-approved fluorescence imaging systems along with their operating parameters. Let's focus on some technical parameters of the presented systems on the market. We believe such information will be helpful, particularly to bioengineers responsible for acquiring and modernizing medical centers.

The VS3 – Visionsense Stereoscopic High Definition (3DHD) Vision System eliminates traditional endoscopic systems' limitations by allowing the clinician to judge depth, volume, or distance accurately. In addition to traditional endoscopic procedures, the VS3 system includes support for IR Fluorescence visualization. The VS3 system is positioned 20–40 cm above the patient during the surgical procedure. VS3 Iridium modules are designed to work with IR fluorescent dyes: ICG. Exciting radiation is directed to the surgical field to excite dye molecules and is subsequently captured using an IR camera [15]. The system allows you to capture a white light image in parallel with a fluorescent IR image and display both images to the surgeon to provide a better image for the surgeon.

The use of a new fluorescent navigation system LIGHTVISION, ICG used for biopsy of the sentinel lymph node in patients with skin cancer, is described in work [16]. LIGHTVISION is a device that comprises a NIR camera with a telescopic arm and a high-resolution monitor. The NIR camera is equipped with a light-emitting diode (780–800 nm) and white light sources; it also possesses NIR and optical image sensors. Routinely, Sentinel lymph node (SLN) biopsy is performed using two methods: blue dye injection (dye method) and radioisotope colloid injection. NIR imaging algorithms are computational processes designed to analyze and interpret data obtained from imaging systems that utilize near-infrared light. These algorithms play a crucial role in extracting meaningful information from the fluorescence signals emitted by contrast agents or fluorophores in biological tissues. They are essential for enhancing the accuracy and efficiency of imaging techniques, particularly in medical applications such as fluorescence-guided surgery and molecular imaging. A novel technique for lymphatic navigation included ICG fluorescence imaging in combination with other techniques.

**Fig. 4:**
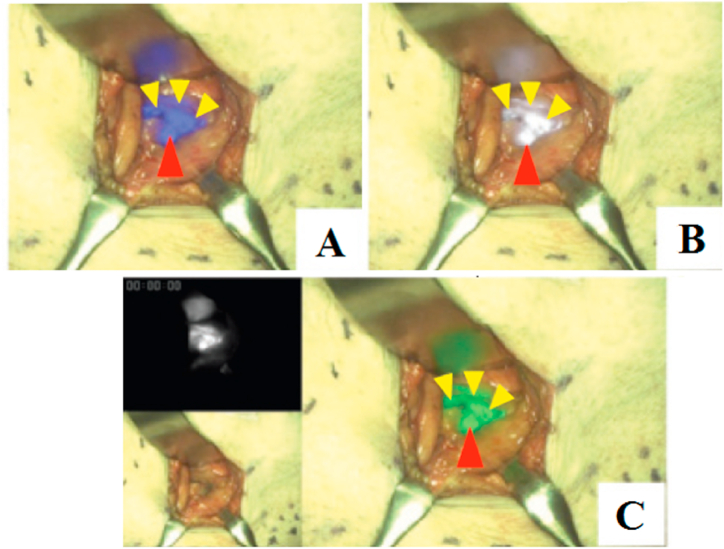
Intraoperative use of LIGHTVISION for detection of sentinel nodes. C) The NIR fluorescent image, visible image, and visible + NIR fluorescent fusion image (right panel); red arrowhead: fluorescent external iliac SLN; yellow arrowheads: solitary and aggregated lymphatics + D, E. Fluorescent pelvic SLN (red arrowhead) and lymphatics (yellow arrowheads) are displayed in blue (D) and white (E) [16]. (For interpretation of the references to color in this figure legend, the reader is referred to the Web version of this article.)

Three patients underwent lymphoscintigraphy via intradermal injection of technetium-99-tin colloid around the primary tumour, the day before surgery. One patient did not receive a radioisotope colloid injection [[16], [17], [18], [19]]. In Fig. 4 [16], after a skin incision over the marked area, nodes are visible, painted in blue; radioactive nodes with an accumulation of radioisotope exceeding a tenth of the maximum value determined by the intraoperative gamma probe; and ICG fluorescent nodes. In total, nine SLNs were detected and excised. Seven radioactive SLNs were detected by preoperative lymphoscintigraphy and intraoperative gamma probe, while two radioactive nodes were negative with the dye method. Conversely, all radioactive nodes showed ICG fluorescence. One ICG-fluorescent SLN was negative based on the dye method and showed no radioactivity (axillary node) [18,19].

ICG binds to albumin and generates a peak wavelength of 840-nm NIR fluorescence when excited with 765-nm light. Using a NIR camera intraoperatively, ICG injected around the primary tumour site can be observed in the subcutaneous lymphatic flow and SLNs on the fluorescence image. The ICG method is beneficial when SLNs cannot be detected using the dye method. By switching the color of the fluorescent image, surgeons can easily distinguish lymphatic vessels and nodes from surrounding tissues. The authors report that it is easier to identify the subcutaneous lymphatic pathways from the overlying skin in the white image than in other highlights. The green image provided better visualization of the differentiation of lymphatic vessels and SLNs from the surrounding adipose tissue. LIGHTVISION has several limitations due to the large size of the main unit and the flexibility of its camera arm.

IMAGE1 S™ Rubina is another technology on the market that combines 3D and 4K imaging with NIR/ICG fluorescence imaging for fluorescence surgery. The RUBINA™ POWER LED cold light source is exclusively based on light-emitting diode technology. Laser-free white light technology and technology for NIR/ICG fluorescent tags in the range or NIR autofluorescence. The system is widely used for a variety of tasks in surgery, namely: multidisciplinary application in laparoscopic, endoscopic, and open surgery; rapid assessment of perfusion of the planned resection area, as well as subsequent anastomosis in the colon; intraoperative visualization of metastases and hepatocellular carcinoma on or under the surface of the liver [20,21].

Fluobeam from FLUOPTICS is another integrated fluorescence imaging solution that provides the surgeon with additional information during surgery, such as tissue perfusion, lymphatic drainage efficiency, and the location of lymph nodes and parathyroid glands. Like similar systems, Fluobeam uses a NIR optical imaging system. However, its optical head is equipped with a class 1 laser as the excitation light source with a NIR sensitive camera. This makes it possible to detect fluorescent markers several millimeters deep into biological tissue. Class 1 lasers are considered harmless to the eyes even when exposed directly. For visible light, the power of the output beam up to 0.39 mW belongs to the safety class for “long-term” visual contact. The term “long-term” visual contact refers to a time greater than ¼ sec. In turn, class 1 is divided into two subclasses: 1 and 1 M. Class 1 M lasers cannot be used with optical instruments such as binoculars or magnifying glasses. The documents provided by the Fluobeam manufacturer do not specify the exact type of laser and do not describe subclassing [22].

## 3 Widely used and clinically available non-specific fluorescence imaging probes

As of 2022, four tumor-targeted probes, namely pafolacianine, BLZ-100, LUM015, and SGM-101, have progressed to phase III clinical trials in the United States [[23], [24], [25], [26], [27], [28]]. Additionally, approximately 40 contrast agents are currently under investigation in more than 85 clinical trials within the United States. Fluorescent tracers generally operate through one of three mechanisms: passive, active, and activatable [24,25]. Passive targeting relies on the enhanced permeability and retention effect (EPR), capitalizing on the heightened vascular permeability and diminished lymphatic drainage characteristic of cancerous tissue. Consequently, the tracer accumulates within the tumor microenvironment. Examples of passive fluorescent indicators include methylene blue, indocyanine green (ICG), 5-Aminolevulinic Acid, and fluorescein [24].

Recently developed tracers actively target abnormally expressed receptors on malignant cells, thereby enhancing labeling specificity and enabling higher resolution imaging. For instance, pafolacianine (OTL38) is a folate receptor α (FRɑ)-targeted tracer that facilitates real-time visualization of radiologically occult ovarian and lung tumors [26,27].

Based on the heightened expression of folate receptor alpha (FRɑ) in lung adenocarcinomas, fluorescent tracers have been chemically linked to folic acid for applications in thoracic surgery. Pafolacianine (OTL38), a near-infrared (NIR) cyanine-based dye conjugated with folic acid, has exhibited efficacy in the identification of positive margins and occult synchronous lesions. In a phase I trial conducted at the University of Pennsylvania, pafolacianine successfully identified all preoperatively identified lung adenocarcinomas (21/21) and demonstrated the ability to detect subcentimeter pulmonary nodules and ground glass opacities. In a subsequent phase II trial involving 110 patients, pafolacianine revealed 24 previously undetected nodules, of which 9 were determined to be cancerous (10 %), and identified 8 positive margins (9 %). These findings resulted in a modification of clinical staging for 7 patients and led to improved outcomes for 26 % of the patients [26].

### 3.1 Indocyanine green (ICG)

Presently, indocyanine green (ICG) stands as one of the extensively utilized near-infrared (NIR) fluorophores in fluorescence-guided surgery (FGS). ICG is characterized as a water-soluble, anionic, amphiphilic tricarbocyanine probe with a molecular weight of 776 Da [29,30], exhibiting rapid binding affinity to the body's plasma proteins. Its excitation peak occurs at 780 nm, while the emission peak is at 820 nm, effectively positioned beyond the typical range of tissue autofluorescence. The substantial overlap between the absorption and fluorescence spectra results in notable fluorescence reabsorption by ICG. The fluorescence spectrum is expansive, with maximum values of approximately 810 nm in water and around 830 nm in blood. In medical applications reliant on absorption, the crucial maximum absorption at approximately 800 nm (in low concentrations of blood plasma) is emphasized. In conjunction with fluorescence detection, lasers with a wavelength of approximately 780 nm are employed [31].

ICG binds strongly to plasma proteins and is restricted to the vasculature. ICG has a half-life of 150–180 s and is eliminated from the circulation exclusively by the liver in bile juice. It is transported into hepatocytes via solute carrier organic anion transporter (SLCO1B3) and sodium-taurocholate cotransporting polypeptide [32] and excreted without transformation into bile mainly via P-glycoprotein 3 (ABCB4) and multidrug resistance protein 2. ICG is often used for navigating tumor sentinel lymph nodes, ophthalmic angiography, selective cell heating, liver function, and gastric blood flow. Due to the intrinsic ability of indocyanine green (ICG) to emit light within the near-infrared (NIR) spectral range, there is minimal interference from background autofluorescence originating from the primary constituents of blood, namely hemoglobin and water. The consequent tissue penetration depth applicable for detecting NIR fluorescence ranges from 0.5 to 1 cm [33]. As highlighted in Ref. [34], autofluorescence poses a significant challenge in pulmonary resections, especially in cases where smoking-induced anthracosis is prevalent.

The utilization of ICG is correlated with a low risk of adverse effects, with the incidence of allergic reactions estimated at one in 42,000 cases. For safety considerations, ICG is contraindicated in individuals with inadequate liver function and those displaying allergies to substances containing iodine. These contraindications, warnings, precautions, and potential adverse reactions are associated with the drug's use. Imaging procedures involve the employment of filtered light sources and highly sensitive charge-coupled cameras capable of detecting probe concentrations at the picomolar and femtomolar levels, offering time resolution ranging from seconds to minutes. ICG lymphography finds active application in preoperative diagnostic procedures, particularly in the surgical management of lymphedema linked to breast cancer [35]. In this preoperative investigation, a subcutaneous injection of 0.1–0.2 mL of ICG dye is administered at the II and IV interdigital web spaces in both hands. The dye is subsequently captured and transported through active lymphatic channels, enabling real-time visualization of fluorescent channels on display. The entire examination is systematically documented. Immediately subsequent to the injection, ICG lymphography reveals an escalating contrast rate towards the axilla. Following a 5-min interval, contrast accumulation occurs, facilitating the assessment of the return flow through the skin. Moreover, this diagnostic approach yields valuable preoperative insights into the extent of damage to the lymphatic system. It not only provides information about the quantity and appearance of augmented lymphatic channels but also delineates the precise location of active lymphatic channels and their transport capacity. The procedure is constrained by its capacity to visualize lymphatics only up to a depth of 2 cm beneath the skin surface. ICG-lymphography holds significant relevance in autologous lymph node transplantation, aiding in comprehending the lymphatic pattern at the donor site limb. In instances where the superficial inguinal nodes serve as donor nodes for transplantation in the axilla, ICG-lymphography, during autologous lymph node transplantation surgery, enables the identification of lymph nodes draining the inferior limb, thereby facilitating their exclusion from the flap.

We would like to draw your attention to the fact that some authors in their works indicate an improvement in the quality of fluorescence imaging in living objects due to detection in the second NIR window (NIR-II, 1000–1700 nm) [36]. This is mainly due to decreased tissue autofluorescence, decreased photon scattering, and low photon absorption at longer wavelengths [[37], [38], [39]].

In the study documented in Ref. [15], the authors delineate an optical imaging apparatus that integrates a visible multispectral imaging system with NIR-I (wavelength 700–900 nm) and NIR-II fluorescence detection, employing indocyanine green dye to facilitate the surgical resection of both primary and metastatic liver tumors under fluorescence guidance in a cohort of 23 patients. Preoperative imaging protocols encompassed enhanced computed tomography (CT), magnetic resonance imaging (MRI), ultrasound, and positron emission tomography (PET). Prior to surgery, patients received intravenous ICG at a dose of 0.5 mg/kg body weight as part of a routine preoperative liver function assessment. Subsequently, one to seven days later, laparotomy was conducted on the day of surgery. Following laparotomy, intraoperative NIR-I/II fluorescence imaging enabled the identification of lesions not detected by preoperative imaging, thereby significantly enhancing the accuracy of staging and overall patient management.

Tumor tissue, normal tissue, or background distinctions can be discerned through NIR-I or NIR-II fluorescence intensity. Comprehensive in vivo and ex vivo imaging studies were conducted. In an in vivo imaging study, the mean tumor area ratio and the tumor-to-normal tissue ratio (TNR) of NIR-II imaging were 2.66 ± 1.14, demonstrating an approximately 2.4-fold increase compared to NIR-I (1.12 ± 0.12). The maximum tumor area and TNR with in vivo NIR-II imaging, along with the tumor-to-background ratio (TBR) in ex vivo NIR-II imaging, were recorded at 5.33 and 5.72, respectively. In contrast, the maximum TNR of in vivo NIR-I imaging and the ex vivo NIR-I imaging TBR were only 1.45 and 2.92, respectively. Both in in vivo and ex vivo imaging studies, a significant enhancement in image contrast ratio was evident in NIR-II as compared to NIR-I. Aligning with the heightened image contrast achieved through intraoperative NIR-II imaging, it also demonstrated a superior detection rate for malignant tumors in comparison to NIR-I (in vivo imaging: 56.41 % vs. 46.15 %; ex vivo imaging of resected specimens: 100 % versus 90.63 %). Article 38 presents a translational investigation that assesses the clinical advantages of near-infrared II (NIR-II) indocyanine green (ICG)-based fluorescence-guided surgery (FGS) in individuals diagnosed with high-grade glioma (HGG). The patients were randomly allocated to either the FGS group or the traditional white light image-guided surgery (WLS) group. The study focused on evaluating the detection rate of NIR-II fluorescence, along with comparisons of complete resection rates, progression-free survival (PFS), overall survival (OS), and neurological status. A total of 15 glioblastoma multiforme (GBM) and 4 WHO grade III glioma patients were enrolled in the FGS group, while 18 GBM and 4 WHO grade III glioma patients were included in the WLS group. The detection rate of NIR-II fluorescence was noted to be 100 % for GBM. FGS significantly increased the complete resection rate for GBM. Additionally, the PFS and OS in the FGS group exhibited significant prolongation. Notably, no recurrence was observed in patients with WHO grade III glioma. This study underscores the viability of employing NIR-II FGS in GBM resection. The NIR-II FGS technique demonstrated a notable achievement in achieving a high 6-month progression-free survival (6m-PFS) and substantially prolonging the PFS and OS of GBM patients.

### 3.2 Methylene blue (MB)

MB is a hydrophobic drug molecule with dual significance as both a staining reagent and a pharmaceutical agent. Possessing strong fluorescence, MB exhibits an emission peak at 686 nm (λex 665 nm) and an additional peak at 293 nm. The molecular weight of the MB compound is 320 Da [40], and it holds FDA approval as a visible (dark blue) contrast agent. When adequately diluted, MB functions as a near-infrared (NIR) fluorescent dye operating within the NIR optical window, featuring an absorption peak at 670 nm, and it is naturally excreted in the urine. Both the FDA and the European Medicines Agency have sanctioned the clinical use of ICG, MB, and 5-aminolevulinic acid (5-ALA) fluorophores. Nevertheless, the clinical efficacy of MB is hampered by its hydrophobic nature, resulting in restricted tissue penetration and heightened autofluorescence from the background tissue. In contrast, ICG exhibits a fluorescence spectrum extending to approximately 800 nm, while 5-ALA possesses a spectrum around 510 nm, both falling outside the NIR fluorescence range [41]. To gain a more comprehensive understanding of the suitability of MB as a fluorescent dye, let us explore specific applications where MB-guided NIR surgical techniques may prove advantageous. One such application pertains to the localization and dissection of parathyroid glands, a challenging aspect of surgical procedures. The identification of enlarged glands poses difficulties due to their variability in number and location. Presently, there are nine parathyroid identification methods available, with five of particular interest in the context of this review: autofluorescence spectroscopy, autofluorescence imaging, ICG imaging, MB fluorescence imaging, and 5-ALA [[40], [41], [42], [43], [44], [45]]. Traditionally, MB is administered intravenously at a high dose (3–7.5 mg/kg) to enhance visibility of the enlarged parathyroid glands, manifesting as a blue stain visible to the naked eye. However, it is crucial to note that these doses, as mentioned earlier, are associated with numerous adverse effects and warrant cautious use [[43], [44], [45]].

**Fig. 5:**
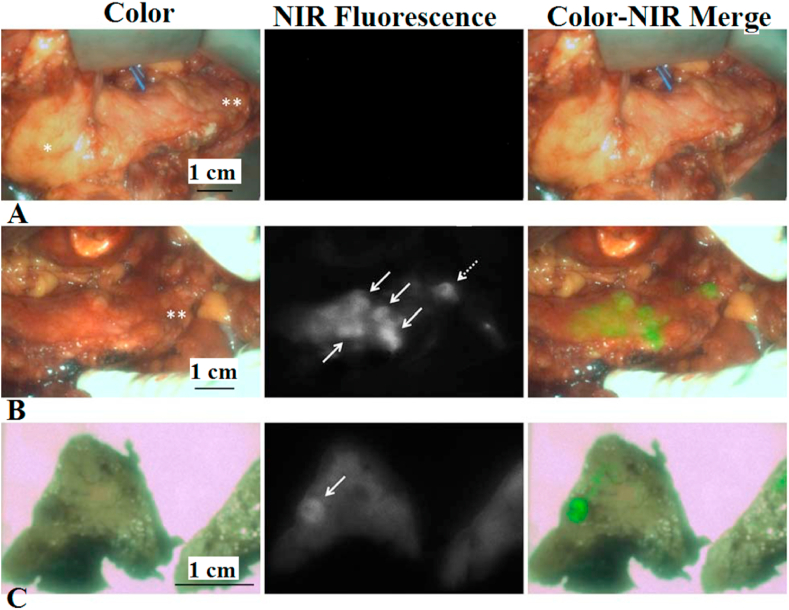
Intraoperative and ex vivo NIR fluorescence imaging of the pancreas. A – Prior to intravenous administration of MB. B – Five minutes after the start of the infusion of MB. Multiple lesions in the pancreas head, body, and tail are fluorescent (exposure time, 50 ms). C – Ex vivo. Faint fluorescence signals remain visible (exposure time, 200 ms) 3 days after resection and formalin fixation. White arrows: lesions suspect for pancreatic neuroendocrine tumors [51].

The NIR fluorescence technique enables the detection of parathyroid glands using reduced doses of MB. According to Hillary et al., the optimal dose for this technique is 0.4 mg/kg, allowing for the effective differentiation of parathyroid glands from the surrounding tissue over a reasonable duration [46]. The lowest intravenous dose of MB administered for distinguishing parathyroid glands from the adjacent thyroid tissue is 0.05 mg/kg. A notable observation from the aforementioned information is the significant reduction in the injection dose of MB during fluorescent analysis, providing a compelling argument in support of its utilization. However, it is essential to acknowledge limitations associated with MB use, including the potential impairment of kidney function as it is excreted by the kidneys. Additionally, the use of MB should be restricted to patients capable of converting MB to non-fluorescent leucomethylene blue due to environmental depletion and/or acidity. Allergic reactions to MB are more frequent at doses exceeding 5 mg/kg; hence, it is advisable to employ the smallest efficacious dose [[47], [48], [49]]. MB finds application in the treatment of neuroendocrine tumors of the pancreas. Intravenous injection of MB results in its accumulation in neuroendocrine tumors, although the precise mechanism behind this action remains unknown [50]. The NIR fluorescence emitted by the pancreas remains relatively stable at a 3.0 signal-to-background ratio for approximately 60 min following intravenous MB injection. The signal-to-background ratio can vary depending on the method of MB administration, with a higher ratio achieved through rapid bolus administration (5–20 s) compared to slow infusion (15–20 min). However, both administration techniques of MB exhibit effective contrast within the pancreas. In cases where multiple endocrine neoplasia is suspected, incomplete resection becomes a possibility [51]. The study outlined in Ref. [51] detailed a case involving insufficient preoperative diagnosis by magnetic resonance imaging, supplemented by positron emission tomography scanning. NIR imaging with MB enabled the visualization of neuroendocrine lesions during surgery, revealing more than 20 lesions that had not been detected by other diagnostic methods across the entire pancreas. This significant finding prompted a change in the surgeon's approach, as illustrated in Fig. 5 [51].

### 3.3 5-Aminolevulinic acid (5-ALA)

5-ALA, an endogenous non-proteinogenic amino acid, serves as the initial compound in the porphyrin synthesis pathway. Porphyrins function as conjugate acids of ligands, binding metals to form complexes, typically with metal ions carrying a charge of 2+ or 3+. In the realm of adult patient care, 5-ALA is employed to enhance the visualization of malignant tissue during surgical interventions for malignant glioma (grade III and IV), specifically in neurosurgical procedures [52]. The excitation peak for 5-aminolevulinic acid is 405 nm, and the emission peak is 635 nm. Previous investigations have demonstrated that intraoperative application of this fluorophore can effectively diminish residual tumor volume and enhance progression-free survival in individuals with malignant gliomas [53,54].

In a study conducted by Sybren et al. [55], the potential for isolating and characterizing circulating extracellular vesicles of tumor origin in glioblastoma patients was explored using oral administration of 5-ALA. Glioblastoma (GB), a malignant tumor of the central nervous system, currently necessitates diagnosis through tissue biopsy, obtainable via resection or (stereotactic) biopsy. However, these invasive procedures pose inherent risks to patients. Extracellular vesicles (EVs), small vesicles derived from cells containing miRNAs, proteins, and lipids, are promising candidates for liquid biopsies. GB-derived EVs can be identified in a patient's blood, yet distinguishing them from circulating non-tumor EVs remains challenging. GB patients treated with 5-ALA aid in tumor visualization and maximal resection, as the substance metabolizes into the fluorescent protoporphyrin IX (PpIX), accumulating in glioma cells.

Concerns have emerged regarding the impact of tumor biopsy on tumor pathophysiology, with studies indicating increased proliferation and migration of tumor cells post-biopsy, and in certain instances, accelerated tumor growth [56,57]. The current unjustifiability of repeated biopsies for follow-up or research purposes is exacerbated by the escalating morbidity and mortality associated with successive biopsy procedures. Conventional imaging modalities like Computed Tomography, Magnetic Resonance Imaging, and Positron Emission Tomography encounter challenges in distinguishing true tumor progression from pseudoprogression. One strategy to meet the need for longitudinal tumor sampling while mitigating patient risks involves the utilization of liquid biopsies [58].

**Fig. 6:**
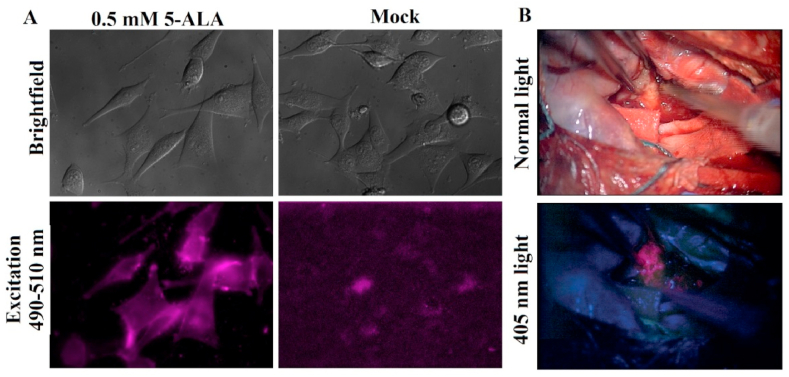
(A) U87 cells treated with 5-ALA (left panels) show PpIX fluorescence upon excitation at 490 nm compared to control (right panels). 40х magnification, (B) Intra-operative view of Glioblastoma resection under white light (top row) and 405 nm light (bottom row). PpIX is seen as pink (fragment of Fig. 1 out of [55]). (For interpretation of the references to color in this figure legend, the reader is referred to the Web version of this article.)

The administration of (5-ALA to a patient pre-surgery allows for uptake by glioma cells, undergoing mitochondrial metabolism to PpIX [59]. Subsequently, PpIX accumulates in glioma cells due to decreased ferrochelatase levels and selective uptake by the ATP-binding cassette transporter ABCB6 [60]. Upon excitation with 405 nm wavelength light, the heightened levels of PpIX in glioma cells induce bright violet-red fluorescence, facilitating the identification of malignant tissue during surgery and enhancing the potential for maximal surgical resection (refer to Fig. 6) [55]. Employing advanced high-resolution flow cytometric sorting enables the isolation of PpIX-positive EVs from the plasma of GB patients. Utilizing digital droplet PCR allows the detection of tumor-specific micro-RNAs in as few as five sorted PpIX-positive EVs. These findings underscore the promise of extracellular vesicle-based fluorescent fluid biopsy in glioblastoma patients. The authors posit that these data demonstrate the feasibility of isolating PpIX-positive glioblastoma-derived EVs from plasma following the oral administration of 5-ALA using high-resolution flow cytometric sorting. However, the utilization of 5-ALA has been hampered by its relatively high costs and the elevated risk of skin sensitization within 24 h post-operation, necessitating patient avoidance of sunlight or intense artificial light exposure [61].

### 3.4 Sodium fluorescein (NaFl)

NaFl is a biologically safe fluorescent dye that extravasates through disruptions in the blood-brain barrier (BBB) [[62], [63], [64]]. Intravenous administration of this drug enhances the visualization of brain tumor tissue, primarily relying on non-specific vascular leakage for imaging. Fluorescein sodium, an organic fluorescent dye and sodium salt, exhibits a peak excitation at 494 nm and a peak emission at 512 nm. Since the 1940s, NaFl has been utilized as an adjunct in the resection of intracranial gliomas and is deemed safe. The reported frequency of adverse reactions ranges from 1 % to 6 % [65]. Notably, a higher incidence of adverse reactions was observed with oral administration compared to intravenous administration. However, when considering the number of usage instances, there was no observed increase in the reports of adverse reactions. While an isolated case of sudden death due to anaphylactic shock was documented among adverse reactions, it is attributed to individual intolerance [66].

Furthermore, NaFl finds active use in ophthalmology for retinal angiography, owing to its relatively lower cost compared to 5-ALA [67]. NaFl is typically discernible to the naked eye at higher dosages (20 mg/kg body weight). Its visibility is enhanced through the yellow 560 nm lower-dose filter, facilitating improved tissue discrimination with more natural colors [62,68,69].

**Fig. 7:**
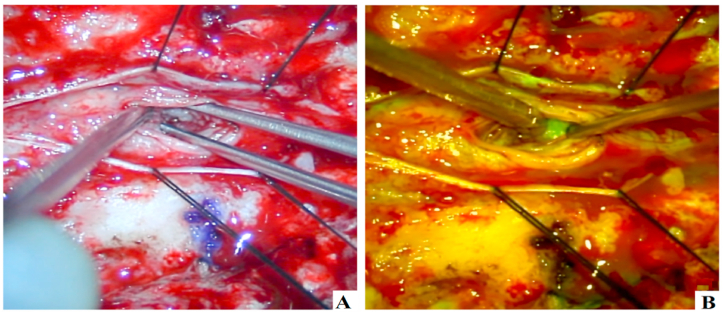
Intraoperative NaFl microscopic images demonstrating lesional fluorescein during microscopic dissection and biopsy (A and B) [70].

Instances of intraoperative NaFl utilization for diagnostic tissue biopsy in spinal cord lesions are documented in Ref. [70]. Three patients, aged 55, 51, and 68 years, who had undergone conservative treatment, underwent surgery for neoplasm biopsy due to persistent clinical symptoms, including unilateral or bilateral limb weakness. In the case of a 55-year-old female patient experiencing progressive back pain, NaFl was administered during a skin incision at a dose of 3 mg/kg. Employing microscopic fluorescein guidance facilitated the easy identification of the lesion, enabling the acquisition of biopsies (see Fig. 7a and b) [70].

The cases presented in Ref. [70] substantiate the utility of NaFl in the resection and biopsy of spinal lesions, encompassing high-grade gliomas, pilocytic astrocytoma, and ependymoma. In these patients, NaFl proved valuable in localizing pathological tissue. Final pathology revealed gram-positive bacteria/intramedullary abscess, EBV-driven B-cell lymphoproliferative disease, and a primary glial neoplasm. Distinguishing abnormal tissue from spinal cord parenchyma can pose challenges for attending surgeons, and the authors assert that NaFl was exceptionally beneficial in identifying abnormal tissue. Thus, NaFl serves as a valuable tool for delineating the resection area and sampling intramedullary and internal spinal lesions when complete resection is not the primary objective.

## 4 New fluorescent agents of molecular action

Fluorescent dyes, which we discussed in the previous section, usually do not have tumor specificity by themselves, so fluorescent agents with molecular action need to be used. So-called tracers consist of a fluorophore chemically conjugated to a targeting moiety, the latter having a binding affinity for a specific cancer molecular target or biomarker. Target tracers are “always active” and “activated” and/or “smart” probes. Tracers that always fluoresce are called “always active” target tracers [13,71]. Smart probes do not fluoresce until activated on the target, suppressing non-specific fluorescent signals and increasing tumor/background contrast [72]. However, fluorescence studies together with computer vision systems, artificial intelligence and telesurgery for the evaluation of pathomorphological images do not dispense the diagnostic gold standards of benign or malignant tumors (conventional examination, biopsy, ultrasonography, and computed tomography, etc.) [[72], [73], [74], [75], [76], [77], [78]].

In this connection, the work [79] is of interest. The authors of the work used a recombinant Helicobacter pylori membrane protein (HopQ) conjugated with the IR800DyeCW fluorophore to target carcinoembryonic antigen-related cell adhesion molecules (CEACAM) – expressing colorectal tumors. It should be noted that the presented results were obtained only on small animals (mice) and require additional studies before clinical use. Previously, carcinoembryonic antigen (CEA) has been identified as an optimal target for in vivo imaging, specifically focusing on colorectal tumors. CEA, also known as CEACAM5 or CD66e, is a cell-surface glycoprotein anchored by glycophosphatidyl inositol, and it is found to be overexpressed in the majority of colorectal cancers [80]. Recent studies have demonstrated that Helicobacter pylori exhibits specific binding to human CEACAM1, 3, 5, and 6 through its outer membrane protein HopQ [81,82]. The interaction between HopQ and CEACAM appears to be independent of calcium and pH, playing a crucial role in facilitating the translocation of the oncogenic effector protein CagA into human host cells [83].

Neoplasm imaging was performed using the LI-COR Pearl Trilogy Small Animal Imaging System (LI-COR, Lincoln, Neb.), which is equipped for fluorescence imaging at 700 and 800 nm. We want to draw readers' attention, especially bioengineers involved in developing and improving systems for fluorescence surgery, to the “chips” used in this system. In the near IR spectrum, autofluorescence of tissues is significantly reduced, making it possible to see tissues located deep inside the animal's body in this case. The LI-COR Pearl Trilogy uses the Pearl Trilogy, which incorporates NIR laser light with a filter system to produce “transparent fabrics”. This leads to sharp light excitation within narrow wavelengths, causing minimal diffusion across the imaging field (CVs <3 %). The system manufacturer, in turn, offers an interesting solution, namely the use of two spectrally different dyes for target marking with fluorescence registration by two channels at 700 and 800 nm. A negative factor can be the image saturation in areas with high signal intensity, after which the data is no longer coherent. Imagers with limited dynamic ranges are unable to simultaneously detect signals differing more than 3 or 4 logs (1000–10,000X) [84].

**Fig. 8:**
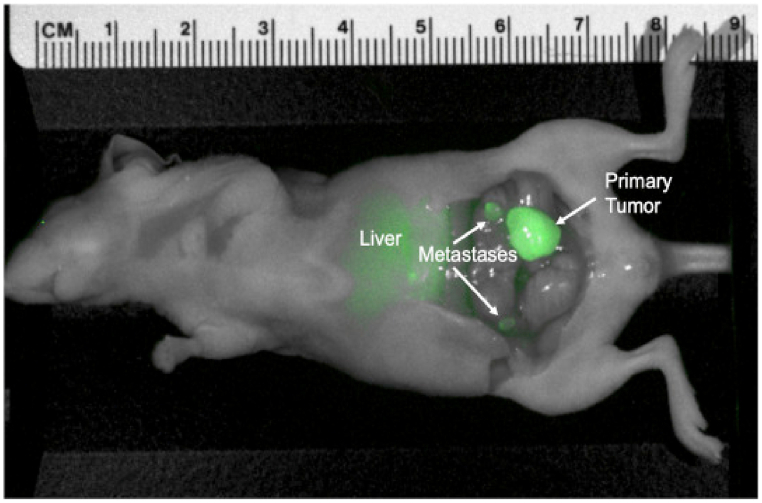
Representative image of orthotopic colon cancer cell-line (LS174T) model, 48 h after administration of 50 μg rHopQ-IR800 [79].

However, let us revisit the outcomes obtained in the subsequent study. Orthotopic models of colon cancer cell lines (n = 6) were subjected to imaging 48 h post-administration of 50 μg rHopQ-IR800. Tumor margins were clearly delineated with a mean tumor-to-background ratio (TBR) = 3.678 (SD ± 1.027). The LS174T orthotopic colon cancer cell-line model exhibited regional metastases measuring approximately 2 mm in diameter, which were distinctly visualized through fluorescence imaging, whereas they remained invisible otherwise (Fig. 8) [79]. Simultaneously, the rHop-IR800 TBR value of 3.678 represents the most favorable outcome when compared to the authors' previous studies, where CEACAM 6G5j-IR800 yielded a TBR of 3.17 [85]. Notably, rHopQ retains its binding capacity to CEACAM1 [83], even when conjugated with various fluorochromes or biotin. Consequently, rHopQ finds applicability in diverse methodologies, including flow cytometry, ELISA, western blotting, and immunohistochemical approaches. Unlike glycosylated antibodies, rHopQ, being a bacterial protein without glycosylation, is protected from degradation.

The examined study underscores the robust binding affinity of rHopQ to epithelial cancers that overexpress CEACAM, encompassing colon, gastric, and lung cancers. Fluorescence imaging facilitated by recombinant HopQ offers distinct visualization of tumor margins in both primary tumors and metastases within patient-derived orthotopic xenograft and orthotopic cell-line mouse models of colon cancer. This suggests that rHopQ could serve as a valuable alternative to CEACAM-specific antibodies for pre-surgical diagnosis, intra-operative imaging, and fluorescence-guided surgery [[83], [84], [85]].

The authors of article [86] sought to assess the discriminatory capacity of a CEACAM5-targeted probe in detecting colorectal cancer (CRC) and to evaluate the utility of NIR-II imaging-guided resection in CRC. The article highlights that conventional fluorescence-guided surgery primarily utilizes the first window (NIR-I, 700–900 nm), characterized by a limited tissue penetration depth ranging from 1 to 6 mm [[87], [88], [89]]. Conversely, the second near-infrared window (NIR-II, 1000–1700 nm) significantly surpasses the constraints imposed by tissue absorption, autofluorescence, and photon scattering, enabling profound tissue penetration (up to 20 mm), micron-scale spatial resolution, intelligent decision-making with U-Net Segmentation, and a high tumor-to-normal tissue (T/NT) ratio exceeding 190 [[90], [91], [92], [93], [94], [95], [96], [97]]. These features hold promise for enhancing T/NT ratio and delineating tumor margins more precisely, thereby facilitating more accurate tumor resection. In this investigation, the authors conjugated an anti-CEACAM5 (2D5) nanobody [97] to the near-infrared fluorescent dye IRDye800CW, resulting in an NIR-II targeted probe: 2D5-IRDye800CW. The purpose was to explore the efficacy of NIR-II fluorescence in intraoperative navigation for colorectal cancer. CEACAM5, overexpressed in 90 % of CRC cases, exhibits an approximately 60-fold lower expression in normal tissue compared to tumor tissue, rendering it an optimal imaging target for CRC [98,99].

**Fig. 9:**
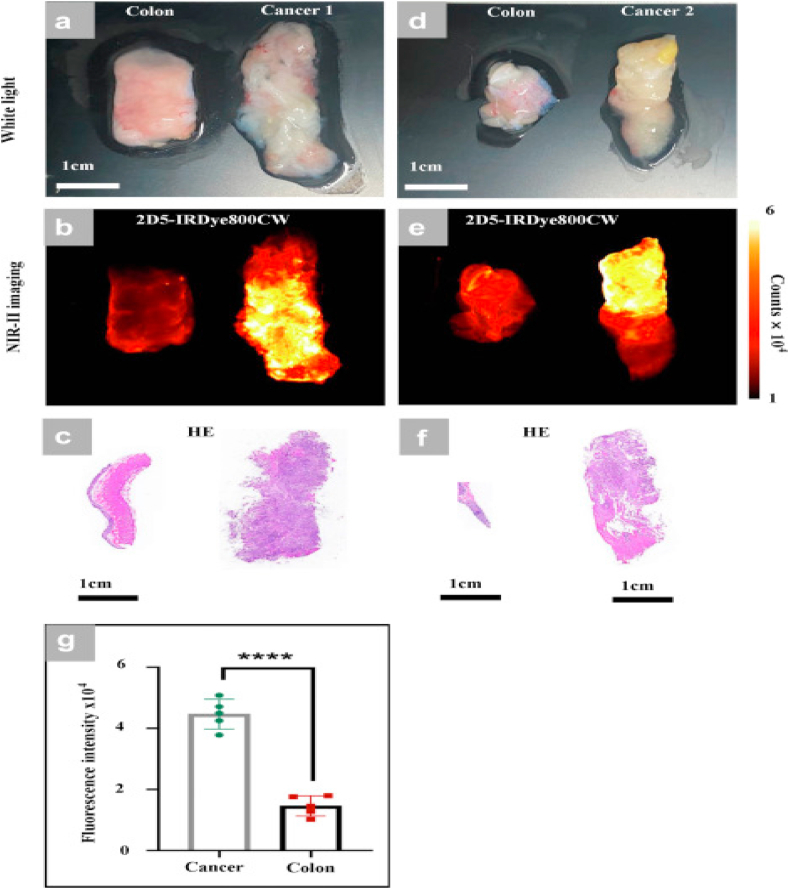
Human colorectal cancer specimens incubated with 2D5-IRDye800CW. (a, d) White light image of isolated colorectal cancer tissue and adjacent normal colorectal tissue. (b, e) NIR-II image after incubation with 2D5-IRDye800CW. (c) Pathological HE staining results (a). (f) Pathological HE staining results (d). (g) Fluorescence intensity of the tumour and adjacent intestinal tissue. Statistical analysis (Student's t-test) with P < 0.05 (∗) was regarded as statistically significant [86].

To assess the imaging efficacy of NIR-II 2D5-IRDye800CW, non-invasive imaging of mouse scalp vasculature was conducted through the intact mouse scalp and skull. NIR-I imaging at 850 nm revealed only large, indistinct vessels following intravenous administration of 2D5-IRDye800CW. Conversely, NIR-II imaging at longer wavelengths (1000–1300 nm) enabled the observation of three distinct blood vessels. Quantitative analysis of the cross-sectional fluorescence intensity distribution demonstrated that, as the wavelength increased from 850 nm to 1300 nm, the peak of NIR-II surpassed that of NIR-I. Similar results were obtained in NIR-I/II fluorescence imaging of hindlimb blood vessels in mice. The study details the authors' execution of R0 colorectal cancer resection under NIR-II fluorescent guidance. An orthotopic mouse model of colorectal cancer was established using HT29-Luc cells (n = 10). Tumors were resected 24 h after the administration of 2D5-IRDye800CW under both white light and NIR-II fluorescence guidance. During the procedure, the abdominal cavity of mice was incised using tissue shears, fully exposing the intestinal tract and peritoneum. Distinguishing normal tissue from tumor tissue under white light was challenging. NIR-II fluorescence imaging revealed a stronger fluorescence signal in the cecum and peritoneum compared to normal tissue. Subsequently, NIR-I imaging signal and bioluminescence imaging (BLI) signal were collected on the small animal living imager. Co-localization of NIR-I/II imaging and BLI signal indicated that areas exhibiting a high fluorescence signal corresponded to tumor tissue. The primary lesion was then excised under the guidance of NIR-II fluorescence, and the peritoneum, showing a high fluorescence signal area, was also excised. NIR-II imaging post-excision revealed no areas with a high fluorescence signal. BLI imaging on postoperative mice showed no high signal areas in the abdominal cavity, confirming the absence of residual tumor tissue and the achievement of R0 resection. The article further details the collection of 5 cases of endoscopic colorectal cancer biopsies and 10 cases of fresh colorectal cancer specimens obtained during surgery. Following incubation with 2D5-IRDye800CW, endoscopic biopsy specimens exhibited high fluorescence intensity in 4 out of 5 cases, along with positive immunohistochemical results for CEACAM5. Probe incubation results for specimens resected during surgery indicated that the fluorescence intensity of tumor tissue in the same patient was significantly higher than that of adjacent normal intestines (Fig. 9 a–f), and this difference was statistically significant (Fig. 9 g) [86]. The presented study [86] underscores the imaging capabilities of 2D5-IRDye800CW at 1000 nm–1500 nm, showcasing deeper tissue penetration and higher spatial resolution, advantageous for intraoperative imaging of deeper tissues and fine blood vessels. However, it is essential to acknowledge that the imaging time of 2D5-IRDye800CW in NIR-II involves a trade-off, as the exposure time at 1000 nm is 30 ms, and it increases to 2 s when imaging at 1500 nm.

## 5 Conclusion

The results of the insertion of fluorescence visualization as an auxiliary method to cancer detection and high-risk metastatic lesions in clinical practice have demonstrated enthusiastic results and huge potential. However, intraoperative fluorescence visualization must not be considered as a main diagnostic or treatment method but as an aid to the surgeon. Thus, fluorescence study does not dispense the diagnostic gold standards of benign or malignant tumors (conventional examination, biopsy, ultrasonography and computed tomography, etc.) and can be done usually during intraoperative treatment. Moreover, as fluorescence surgery and fluorescence diagnostic techniques continue to improve, it is likely that they will evolve towards targeted fluorescence imaging probes that will increasingly target a specific type of cancer cell. The most important point remains the search for highly selective messengers of fluorescent labels, which make it possible to identify tumor cells exclusively in the affected organs and indicate to surgeons the boundaries of their spread and metastasis.

### 5.1 Сonflicts of interest

The authors report no conflict of interest. The authors declare that the research was conducted in the absence of any commercial or financial relationships that could be construed as a potential conflict of interest.

## Data availability statement

The datasets generated and analyzed during the current study are available in the Electronic Sumy State University Institutional Repository [https://essuir.sumdu.edu.ua/] and Social Science Research Network [https://papers.ssrn.com/sol3/papers.cfm?abstract_id=4462115]. Additional supporting data are available from the corresponding author upon reasonable request.

## CRediT authorship contribution statement

**Yaroslav Kravchenko:** Writing – review & editing, Writing – original draft, Visualization, Validation, Supervision, Project administration, Methodology, Investigation, Formal analysis, Data curation, Conceptualization. **Kateryna Sikora:** Writing – review & editing, Writing – original draft, Validation, Supervision, Project administration, Methodology, Funding acquisition, Formal analysis, Data curation, Conceptualization. **Andrew Awuah Wireko:** Writing – review & editing, Writing – original draft, Visualization, Validation, Software, Methodology, Conceptualization. **Mykola Lyndin:** Writing – review & editing, Writing – original draft, Validation, Supervision, Resources, Project administration, Methodology, Funding acquisition, Formal analysis, Data curation, Conceptualization.

## Declaration of competing interest

The authors declare the following financial interests which may be considered as potential competing interests:Mykola Lyndin reports financial support was provided by 10.13039/501100007684Ministry of Education and Science of Ukraine. Yaroslav Kravchenko reports financial support was provided by NCN OPUS project. If there are other authors, they declare that they have no known competing financial interests or personal relationships that could have appeared to influence the work reported in this paper.
